# The effect of gestational weight gain on the infant gut microbiome- a systematic review of the literature

**DOI:** 10.3389/fcimb.2026.1751708

**Published:** 2026-02-10

**Authors:** Nikoleta Aikaterini Xixi, George Karamanolis, Evgenia-Eleni Vlachogianni, Theodoros Voulgaris, Rozeta Sokou, Paraskevi Volaki, Styliani Paliatsiou, Zoi Iliodromiti, Nicoletta Iacovidou, Theodora Boutsikou

**Affiliations:** 1 Neonatal Department, Aretaieio Hospital, School of Medicine, National and Kapodistrian University of Athens, Athens, Greece; 2 Endoscopy Department, 2nd Academic Surgical Unit, National and Kapodistrian University of Athens, Aretaieion Hospital, Athens, Greece; 3 School of Medicine, National and Kapodistrian University of Athens, Athens, Greece

**Keywords:** infant gut microbiota, microbiome, obesity, overweight, weight gain

## Abstract

**Introduction:**

Maternal weight status and gestational weight gain (GWG) critically affect maternal and neonatal health. The infant gut microbiome is a key predictor of short- and long-term child health. Therefore, investigating how maternal weight characteristics influence the composition and establishment of the infant’s gut microbiome is essential.

**Objective:**

To evaluate the impact of excessive GWG on the infant gut microbiome.

**Methodology:**

PubMed and Scopus were systematically searched for studies on GWG from September 1^st^ until October 1^st^, 2025. Data on infant gut microbiome characteristics and their relation to maternal weight change during pregnancy were extracted. The systematic review is registered in PROSPERO (CRD 420251181399).

**Results:**

A total of 15 studies met the inclusion criteria and were included in this review. Excessive Gestational Weight Gain (EGWG) consistently appeared to impair infant gut microbial alpha diversity, an effect that persisted up to 12 months. Taxonomically, EGWG caused a shift away from beneficial *Bacteroides* toward opportunistic/pathogenic genera (e.g., *C. difficile*). The negative effects are synergistically exacerbated by co-occurring Gestational Diabetes Mellitus (GDM) and are functionally characterized by an independent shift toward microbial carbohydrate degradation and vitamin synthesis pathways. Clinically, this EGWG-induced dysbiosis is linked to increased early childhood weight gain.

**Conclusion:**

EGWG is an independent, critical determinant of persistent infant gut dysbiosis, characterized by taxonomic and functional shifts. These findings establish EGWG as a key modifiable maternal factor, linking gestational health to infant gut microbiome and health.

**Systematic Review Registration:**

https://www.crd.york.ac.uk/PROSPERO/view/CRD420251181399, identifier: CRD 420251181399.

## 1 Introduction

Increased body weight is one of the most important global health issues, affecting over 2 billion people worldwide (Engin, 2024). Maternal obesity creates a bad intrauterine environment and as a consequence may have adverse effects and a negative impact on the mother during pregnancy and on the infant in the beginning and later in life (Anstey et al., 2011).

Gestational weight gain (GWG) is a crucial physiological adaptation that occurs in response to the increased metabolic demands of the growing fetus. According to the Institute of Medicine (IOM) recommendations, for singleton pregnancies, the recommended total weight gain is approximately 12.5–18 kg for women who enter pregnancy underweight (BMI <18.5), 11.5–16 kg for those of normal BMI (18.5-24.9), 7-11.5 kg for overweight women (BMI 25-29.9), and 5–9 kg for women with obesity (BMI ≥30). In the context of twin pregnancies, the suggested weight gain thresholds are correspondingly higher: about 16.8-24.5 kg for women with a normal BMI, 14.1-22.7 kg for those who are overweight, and 11.3-19.1 kg for women with obesity (Weight Gain During Pregnancy).

Maintaining the appropriate weight gain dynamics is critical, as inadequate or excessive GWG are consistently associated with adverse maternal and neonatal outcomes that can impact long-term health status (Shekaili et al., 2024). Specifically, inadequate prenatal weight gain risks fetal growth restriction and preterm birth, while excessive weight gain (including pre-existing obesity, or obesity developing during gestation) is linked to a higher incidence of prematurity, fetal death, neonatal metabolic disturbances, and increased congenital risks for the child, such as neural tube defects and congenital heart defects (Shekaili et al., 2024; Ege et al., 2025).

While the fetal environment was traditionally considered sterile, some recent research has proposed the possibility of prenatal microbial exposure via the placenta and amniotic fluid (Li et al., 2024). However, this remains a subject of significant debate, with delivery mode and early postnatal factors currently recognized as the primary and most definitive drivers of initial gut colonization, with the surrounding environment, breast milk, feces, mouth, and skin being the primary sources of the first microorganisms for all newborns (Ma et al., 2023). During the initial months of life, the intestinal tract is transiently overpopulated by facultative anaerobes such as Enterobacteriaceae and Staphylococcus, which are quickly replaced by the “Bifidus flora,” a dominant population of Bifidobacterium and lactic acid bacteria that remains stable until the introduction of complementary solid foods. As the infant approaches weaning, the relative abundance of *Bacteroides* increases, gradually replacing *Bifidobacterium* and leading to an adult-type community dominated by *Bacteroides, Prevotella, Clostridium*, and *Ruminococcus*. This shift, typically resulting in an adult-like microbiota by three years of age, is significantly shaped by crucial environmental factors, including breastfeeding, type of delivery, and antibiotic exposure (Pantazi et al., 2023; Li et al., 2024).

Prior reviews suggested a relationship between maternal obesity and both maternal and infant gut microbiome, noting that increased pre-pregnancy body-mass index (BMI) was often linked to early differences in infant gut composition and alpha diversity. However, the authors concluded that the literature was highly heterogeneous, observing contradictory effects for GWG and emphasizing that any microbial differences were often nullified or weakened by postnatal factors like delivery mode and feeding (Grech et al., 2021; Mulligan and Friedman, 2017; Singh et al., 2017; Kumbhare et al., 2019; Dreisbach et al., 2020; Kapourchali and Cresci, 2020; O’Neill et al., 2020). Given that the gut microbiome remains an ever-evolving field of research, constantly revealing new pathways and interactions, there is a critical need to update and narrow the synthesis. Therefore, our review aims specifically to focus solely on the effects of GWG on the infant gut microbiome, in order to provide a more precise and current synthesis regarding this single, critical perinatal variable.

## 2 Materials and methods

This systematic review was conducted according to the Preferred Reporting Items for Systematic reviews and Meta-Analyses (PRISMA) statement (Grainger and , 2025). A prespecified protocol was formulated and registered in PROSPERO (CRD 420251181399) and is available online (PROSPERO, [[NoYear]]).

### 2.1 Search strategy

Two authors (NAX and EEV) independently conducted the literature search, and any discrepancies were solved through discussion. PubMed and Scopus were systematically searched. References of the retrieved articles were also screened for relevant literature. A search phrase including keywords (namely gestational weight gain, maternal obesity, infant gut microbiome, gut microbiota) and Boolean operators was formulated. All relevant literature with no language, time and geographical restrictions from September 1^st^ until October 1^st^, 2025, was retrieved. All observational studies and randomized controlled trials providing collective data in infant gut microbiota, in relation with maternal gestational weight gain were assessed for inclusion to our study. Review articles of any type, meta-analyses, comments, editorials and case reports or case series with less than 5 patients, or studies analyzing the characteristics of gut microbiota beyond the infantile age or only reporting on maternal BMI with no mention on weight gain dynamic changes during pregnancy were excluded from our review.

### 2.2 Data extraction

Data extraction was conducted independently by two authors (NAX, EEV). For the purpose of the data extraction, a prespecified table including data on the name of the first author, publication year, country of research, maternal characteristics (e.g., maternal age, prior antibiotic use, probiotic supplementation, gestational age, pregnancy pathologies or complications, mode of delivery, feeding method), infant characteristics, and key findings related to gut microbiome of the offspring was formulated. Any discrepancies were resolved through discussion between the two authors.

### 2.3 Definitions

The population will consist of infants from birth up to two years of age (24 months), allowing for the capture of both immediate microbial colonization data and longitudinal effects. As we expected to encounter highly heterogeneous populations with varying metabolic and ethnic profiles, GWG classification cut-offs were defined according to the specific definitions and thresholds used within each individual included study.

### 2.4 Outcomes

The primary outcome is the evaluation of microbial diversity and taxonomic composition in the infant gut. Specifically, diversity and richness (alpha-diversity) of the microbial community, alongside community structure (beta-diversity), the relative abundance of specific microbial taxa in the gut microbiome of infants whose mothers gained above the recommended weight during pregnancy will be reported.

## 3 Results

**Figure 1:**
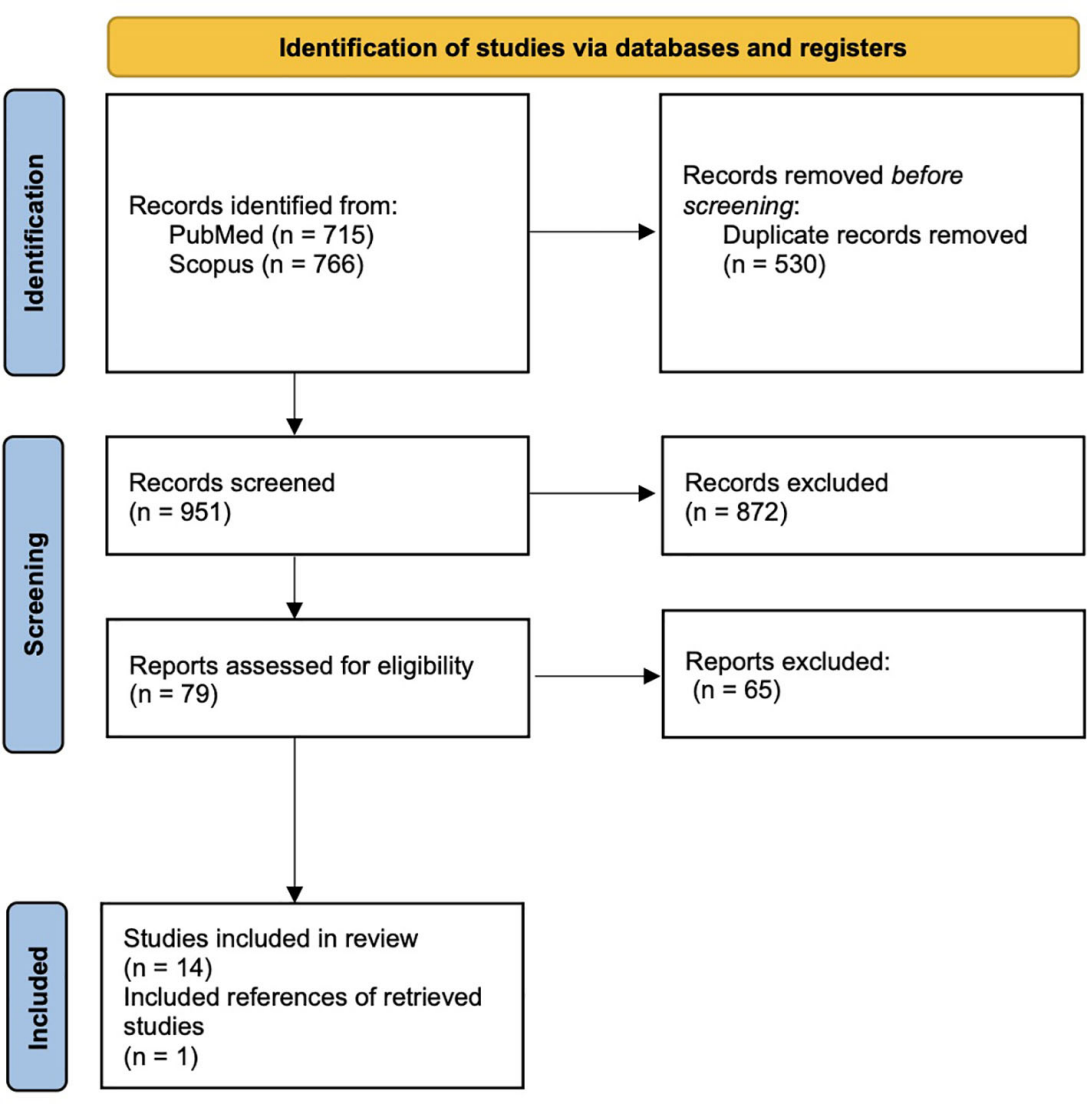
Identification of studies via databases and registers.

A total of 1481 articles were initially retrieved from PubMed and Scopus. Out of the 79 of these who were assessed for eligibility, 14 met the inclusion criteria. One additional study was identified by screening the reference lists of the initially retrieved articles, resulting in a final total of 15 studies included in this systematic review (Kennedy et al., 2023; Collado et al., 2010; Chu et al., 2017; Robinson et al., 2017; Stanislawski et al., 2017; Baumann-Dudenhoeffer et al., 2018; Singh et al., 2020; Raspini et al., 2021; Gilley et al., 2022; Vacca et al., 2022; Song and Liu, 2023; Caprara et al., 2024; Xiao et al., 2024; Cho et al., 2025; Liu et al., 2025). The selection process is presented in the PRISMA flow diagram of the study (**Figure 1**).

The 15 studies were included in this review, yielded approximately 1723 participants. The included studies span a publication period of 15 years, ranging from 2010 to 2025. The studies were conducted across eight countries, with the USA now being the most represented (Chu et al., 2017; Robinson et al., 2017; Baumann-Dudenhoeffer et al., 2018; Singh et al., 2020; Gilley et al., 2022), and including also China (Song and Liu, 2023; Xiao et al., 2024; Liu et al., 2025), Italy (Raspini et al., 2021; Vacca et al., 2022), and one study each in Korea (Cho et al., 2025), Germany (Kennedy et al., 2023), Brazil (Caprara et al., 2024), Norway (Stanislawski et al., 2017), and Finland (Collado et al., 2010). The characteristics of the included studies are summarized in **Table 1**.

**Table 1:** Study characteristics.

First author	Year	Country	Time period	Study design	Infants, N	Aim
Baumann-Dudenhoeffer et al (Baumann-Dudenhoeffer et al., 2018)	2018	USA	NA	Observaitonal cohort study	60	To build a comprehensive map of how maternal and environmental factors in early life shape the developing infant gut and its functional potential.
Caprara et al (Caprara et al., 2024)	2024	Brazil	NA	Cross sectional study	30	To characterize the newborn gut microbiota according to mode of delivery and maternal pre-pregnancy BMI.
Cho et al (Cho et al., 2025)	2025	Korea	2021-2022	Prospective cohort study	71	To explore the relationship between maternal weight categories and the composition of the infant gut microbiome.
Chu et al (Chu et al., 2017)	2017	USA	NA	Prospective cohort study	81	To assess the composition and metabolic function of the neonatal and early infant microbiota and assess the impact of mode of delivery and its potential confounders.
Collado et al (Collado et al., 2010)	2010	Finland	2002	Longitudinal cohort study	42	To evaluate the effects of the pre-pregnancy weight of mothers and GWG on infant microbiota acquisition and development during the first 6 months of life.
Gilley et al (Gilley et al., 2022)	2022	USA	NA	Prospective cohort study	170	To examine infant fecal microbiome, SCFA and maternal HMO in OW mothers compared to NW.
Kennedy et al (Kennedy et al., 2023)	2023	Germany	NA	Prospective cohort study	58	To investigate the impact of maternal pre-pregnancy BMI and GWG on the gut microbiota of both mothers and their infants.
Liu et al (Liu et al., 2025)	2025	China	2018-2019	Case-control study	247	To investigate whether perinatal characteristics affect the association between maternal GDM status and early neonatal gut microbiota.
Raspini et al (Raspini et al., 2021)	2021	Italy	NA	Prospective cohort study	53	To explore the prenatal and postnatal factors influencing the infant gut microbiota composition at six months of age.
Robinson et al (Robinson et al., 2017)	2017	USA	2013-2014	Prospective cohort study	84	Determine associations of maternal GWG with infant fecal microbiota profiles, bacterial community richness, and Shannon diversity index
Singh et al (Singh et al., 2020)	2020	USA	2009	Retrospective cohort study	335	To examine prospective associations of maternal pre-pregnancy BMI and GWG with the infant gut microbiome by delivery-mode strata.
Song et al (Song and Liu, 2023)	2023	China	2020	Cross-sectional study	68	To investigate the association of GWG on gut microbiota in pregnant women and newborns.
Stanislawski et al (Stanislawski et al., 2017)	2017	Norway	2002-2005	Longitudinal cohort study	181	To determine how maternal pre-pregnancy BMI and GWG impact the gut microbiota composition and diversity of mothers at delivery and their infants during the first two years of life.
Vacca et al (Vacca et al., 2022)	2022	Italy	NA	Prospective cohort study	45	To characterize the gut microbiota and determine how different prenatal, perinatal, and postnatal factors affected its composition in early childhood
Xiao et al (Xiao et al., 2024)	2024	China	2021-2022	Prospective cohort study	98	To investigate the combined impact of GDM and excessive gestational weight gain (EGWG) on the neonatal gut microbiota

NA, Not Applicable; GDM, Gestational Diabetes Mellitus; BMI, Body mass index; GWG, Gestational Weight Gain; SCFA, short chain fatty acids; HMO, maternal human milk oligosaccharides; OW, Overweight; NW, Normal weight; N, Number.

### 3.1 Fecal sampling and analysis

**Table 2** summarizes key methodological characteristics of the included studies. 14 out of 15 studies utilized 16S rRNA gene sequencing to characterize the microbiome of the infant gut, showing high homogeneity in that aspect (Kennedy et al., 2023; Chu et al., 2017; Robinson et al., 2017; Stanislawski et al., 2017; Baumann-Dudenhoeffer et al., 2018; Singh et al., 2020; Raspini et al., 2021; Gilley et al., 2022; Vacca et al., 2022; Song and Liu, 2023; Caprara et al., 2024; Xiao et al., 2024; Cho et al., 2025; Liu et al., 2025). Two studies also incorporated Whole Genome Shotgun Sequencing for deeper analysis (Chu et al., 2017; Baumann-Dudenhoeffer et al., 2018), and one older study used qPCR and FCM-FISH (Collado et al., 2010). There was notable variability in the timing of fecal sampling. Meconium samples were frequently collected, sometimes followed by later time points of collection. Other studies focused on longitudinal collection across the first year of life (Stanislawski et al., 2017; Gilley et al., 2022), or specific later windows (Singh et al., 2020; Vacca et al., 2022). Regarding the definition of GWG, the majority of studies relied on the Institute of Medicine (IOM) guidelines, or guidelines derived from IOM recommendations. Other definitions included classifications based on local or established literature (Singh et al., 2020; Xiao et al., 2024), or specific weight categories (Robinson et al., 2017).

**Table 2:** Methodology of the individual studies.

First author	Fecal sampling	Analysis method	GWG definition
Baumann-Dudenhoeffer et al (Baumann-Dudenhoeffer et al., 2018)	Monthly collection from birth to 8 mo.	Whole-Metagenome Shotgun Sequencing	IOM recommendations for twin pregnancies^*^
Caprara et al (Caprara et al., 2024)	Meconium 24–48 h. pp.	16S rRNA sequencing	NA
Cho et al (Cho et al., 2025)	Meconium <5 d. pp.	16S rRNA sequencing	IOM recommendations^*^
Chu et al (Chu et al., 2017)	At birth and at 4–6 w.	16S rRNA sequencing, Whole Genome Shotgun Sequencing	NA
Collado et al (Collado et al., 2010)	At 1 and 6 mo.	qPCR, FCM-FISH	IOM recommendations^*^
Gilley et al (Gilley et al., 2022)	At 1, 6, and 12 mo.	16S rRNA sequencing	IOM recommendations^*^
Kennedy et al (Kennedy et al., 2023)	At 6 mo.	16S rRNA sequencing	IOM recommendations^*^
Liu et al (Liu et al., 2025)	Meconium collected within 24 h pp.	16S rRNA sequencing	NA
Raspini et al (Raspini et al., 2021)	Meconium and 6 mo.	16S rRNA sequencing	IOM recommendations^*^
Robinson et al (Robinson et al., 2017)	Collected by parents	16S rRNA sequencing	three groups: ≤11.9 kg, 12.0–14.9 kg, and ≥15.0 kg
Singh et al (Singh et al., 2020)	6 w. pp.	16S rRNA sequencing	Gilmore and Redman’s method^**^
Song et al (Song and Liu, 2023)	Meconium pp;	16S rRNA sequencing	EGWG: > 16 kg, > 11.5 kg and > 9 kg in normal weight, overweight women.
Stanislawski et al (Stanislawski et al., 2017)	d. 4, 10, 30, 120, 365, and 730 pp.	16S rRNA sequencing	IOM recommendations^*^
Vacca et al (Vacca et al., 2022)	12 mo.	16S rRNA sequencing	IOM recommendations^*^
Xiao et al (Xiao et al., 2024)	24 h. pp.	16S rRNA sequencing	EGWG: GWG >14 kg

NA, Not Applicable; GWG, Gestational Weight Gain; IOM, Institute of Medicine; mo, months; h, hours; d, days; pp, post-partum; w, weeks.

^*^11.5–16 kg for normal weight pre-pregnancy (NW), 7–11.5 kg for overweight pre-pregnancy (OW), and 5–9 kg for obesity pre-pregnancy (OB), and 16.8–24.5kg for NW, 14.1–22.7 kg for OW, 11–3-19.1 kg for OB (American College of Obstetricians and Gynecologists, 2013).

^**^0.36−0.45 kg/wk for NW, 0.23−0.32 kg/wk for OW, 0.18−0.27 kg/wk for OB (Gilmore and Redman, 2015).

### 3.2 The impact of EGWG on alpha diversity and community richness

**Table 3** summarizes the key findings of the included studies. A consistent finding is the negative relationship between EGWG and the overall richness and diversity of the infant gut microbiota. Higher rates of GWG were found to significantly impair the vertical transmission and establishment of a diverse microbial community. Robinson et al. demonstrated that increased maternal GWG was negatively correlated with both bacterial community richness (Spearman’s ρ=-0.25, P = 0.02) and the Shannon diversity index (Pearson’s ρ =−0.25, P = 0.02) (Robinson et al., 2017). The same effect was also observed by Kennedy et al., where maternal GWG (overall categories, including EGWG) significantly impacted infant alpha diversity (number of observed ASVs) (p=0.047) (Kennedy et al., 2023).

**Table 3:** Summary table of individual study findings.

First author	Taxa	Study findings
Baumann-Dudenhoeffer et al (Baumann-Dudenhoeffer et al., 2018)	*Bifidobacteriaceae, Lachnospiraceae, Enterobacteriaceae, Bacteroidaceae*	eight-month shift toward microbial pathways for carbohydrate degradation and vitamin synthesis related to GWG
Caprara et al (Caprara et al., 2024)	*Prevotella, Rothia, Mycoplasma, Fusobacterium*	EGWG lead to lower alpha diversity.
Cho et al (Cho et al., 2025)	*Prevotellaceae, Lachnospiraceae, Bifidobacteriaceae, Moraxellaceae, Rhodospirillaceae, Burkholderiaceae, Peptococcaceae, Sphingomonadaceae, Sphingobacteriaceae, Bacillaceae, Alteromonadaceae, Cytophagaceae, Enterococcaceae, Erysipelotrichaceae*	EGWG - higher alpha diversity in the meconium - Beta Diversity: No significant differences *Klebsiella*: higher in the Inadequate GWG group compared to the Excessive GWG group (p<0.05). *Holdemania*: highest in the Adequate GWG group, compared to both Inadequate and Excessive GWG groups (p<0.05).
Chu et al (Chu et al., 2017)	At Delivery (Meconium): *Escherichia, Klebsiella Lactobacillus, Bifidobacterium, Bacteroides, Propionibacterium, Streptococcus, Staphylococcus.* At 6 Weeks (Stool): *Escherichia, Klebsiella, Lactobacillus, Bifidobacterium.*	GWG - not a significant contributor to the abundance of *Bacteroides*, *Bifidobacterium*, or *Lactobacillus* - little impact on the variation of functional pathways (metagenome) within the infant stool.
Collado et al (Collado et al., 2010)	*Bifidobacterium (B. longum, B. breve, B. bifidum, B. adolescentis, B. catenulatum), Clostridium (Cl. Leptum, Cl. Perfringen,s Cl. Difficile), Staphylococcus aureus, Akkermansia muciniphila*	EGWG: - *Clostridial/Firmicutes* increase at 6 months of age - Protective *Bifidobacteria* decrease at 6 months of age - *Bacteroides* decrease at 1 month of age - Pathogen/Inflammatory Indicator Increase
Gilley et al (Gilley et al., 2022)	*Proteobacteria, Bacteroidetes, Firmicutes, Verrucomicrobia*	EGWG: - Adverse relation to *Akkermansia* abundance - Alpha Diversity: EGWG was positively associated with Chao1 index at 6 months.
Kennedy et al (Kennedy et al., 2023)	*Lactobacillaceae, Bifidobacteriaceae, Enterobacteriaceae, Ruminococcaceae, Veillonellaceae, Lachnospiraceae, Corynebacteriales, Flavobacteriaceae, Erysipelotrichaceae, Bacteroidaceae*	EGWG: - Alpha Diversity: Maternal GWG significantly impacted infant alpha diversity - Beta Diversity: EGWG showed a significant effect on infant beta diversity only in infants born to multiparous participants - Taxonomic Shifts: Increased abundance of *Bifidobacterium*
Liu et al (Liu et al., 2025)	*Firmicutes, Proteobacteria, Bacteroidetes, Actinobacteria*	Appropriate and excessive GWG caused significant differences in the neonatal gut microbiota, and this influence was stronger in the Control group than in the GDM group.
Raspini et al (Raspini et al., 2021)	*Ruminococcaceae, Clostridiaceae, Peptostreptococcaceae, Erysipelotrichaceae Incertae Sedis, Clostridiales Incertae Sedis XIII, Staphylococcaceae, Cellulomonadaceae, Corynebacteriaceae, Actinomycetaceae, Streptomycetaceae, Micromonosporaceae, Colwelliaceae.*	EGWG: - No Alpha Diversity Impact - No Significant Taxonomic Differences
Robinson et al (Robinson et al., 2017)	*Escherichia, Bifidobacterium, Enterobacter, Bacteroides*	- Negative relationship between GWG and likelihood of *Bacteroides*-dominant profile. - Negative correlations of GWG with richness and Shannon diversity.
Singh et al (Singh et al., 2020)	*Bacteroides, Parabacteroides, Staphylococcus, Escherichia Enterococcus, Klebsiella, Ruminococcus, Dorea, Bifidobacterium*	EGWG: - Diversity (Alpha/Beta): No association - In vaginally-delivered infants a higher relative abundance of two genera (compared to Adequate GWG) - In Cesarean-delivered infants, Excessive GWG was associated with a lower relative abundance of *Dorea*.
Song et al (Song and Liu, 2023)	*Lactobacillaceae, Bifidobacteriaceae, Enterobacteriaceae, Ruminococcaceae, Veillonellaceae, Lachnospiraceae, Corynebacteriales, Flavobacteriaceae, Erysipelotrichaceae, Bacteroidaceae*	EGWG: - Alpha Diversity Reduction - Beta-Diversity: The Normal PBMI + EGWG group was clearly distinguishable from all other groups Phylum-Level Shifts - irmicutes: Higher relative abundance in the EGWG group - *Bacteroidetes*: Lower relative abundance in the EGWG group - *Actinobacteria*: Lower relative abundance in the EGWG group Taxa Associated with - Normal PBMI + EGWG: A significant number of genera, including *Aeromonas*, *Staphylococcus*, *Lactobacillus*, *Ruminococcaceae*, and *Pseudomonadaceae*, suggesting an increase in aerotolerant anaerobes. - PBMI + EGWG: Taxa included *Bifidobacteriaceae*, *Enterobacter*, and *Veillonellaceae*.
Stanislawski et al (Stanislawski et al., 2017)	*Lachnospira, Parabacteroides, Bifidobacterium, Blautia, Methanobrevibacter, Bacteroides, Ruminococcus, Faecalibacterium, Finegoldia*	EGWG was not a significant determinant of the infant gut microbiota’s alpha diversity or overall taxonomic composition over the first two years of life.
Vacca et al (Vacca et al., 2022)	*Ruminococcaceae, Bacteroidaceae, Lactobacillaceae, Enterobacteriaceae, Veillonellaceae, Acidaminococcaceae, Pasteurellaceae, Streptococcaceae*	EGWG: - Higher *Actinobacteria* abundance - Lower *Firmicutes* abundance
Xiao et al (Xiao et al., 2024)	*Actinobacteria, Bacteroidetes, Firmicutes, Proteobacteria*	Highly distinct Beta-diversity between GDM+EGWG and GDM+NGWG infants.

GWG, Gestational Weight Gain; EGWG, Excessive Gestational Weight gain; GDM, Gestational Diabetes Mellitus.

Research showed that EGWG is often not temporary. The study by Gilley et al. revealed that infants born to mothers whose GWG exceeded the IOM recommendations exhibited a significantly lower alpha diversity index that was still measurable at 12 months of age (Gilley et al., 2022). This suggests that the early effects of EGWG establish a less diverse microbiota that is slow to recover. In the study by Stanislawski et al., while GWG was associated with differences in the maternal gut microbiota, it did not lead to overall differences in the infant’s community structure over the first two years of life (Stanislawski et al., 2017).

### 3.3 EGWG and taxonomic composition

#### 3.3.1 Disruption of beneficial *Bacteroides* colonization

Most studies reporting genus-level shifts indicate that higher GWG causes a decreased *Bacteroides* abundance, a genus crucial for immune maturation and short-chain fatty acid (SCFA) production. Collado et al. found that mothers with excessive weight gain during pregnancy gave birth to infants who had lower concentrations of *Bacteroides* at one month of age, a finding which persisted at six months (Collado et al., 2010). Additionally, Robinson et al. reported a negative linear relationship between the overall increase in GWG and the likelihood of an infant having *Bacteroides*-dominant microbiome. Specifically, a 1 Kg increase in GWG correlated with an unadjusted RR of 0.83 (CI, 0.71-0.96, P = 0.01) for having a *Bacteroides*-dominant profile relative to an *Enterobacter*-dominant profile (Robinson et al., 2017). This association remained statistically significant in adjusted models controlling for confounders. On the other hand, Chu et al. noted that after controlling for confounding factors, GWG was not a significant factor contributing to the abundance of *Bacteroides, Bifidobacterium*, or *Lactobacillus* (Chu et al., 2017). Lastly, in the study by Vacca et al, there was a tendency for *Actinobacteria* abundance to be higher in the EGWG group (p=0.021), and for Firmicutes abundance to be lower (p=0.034).

#### 3.3.2 Proliferation of pathogenic and opportunistic taxa

In addition to the reduction in beneficial taxa, EGWG was also associated with the increased presence and of potentially pathogenic or opportunistic bacteria. Collado et al. found that EGWG is linked to an increase in *Clostridium difficile* in infants at six months of age (P = 0.046) and that the *Staphylococcus aureus* group was detected more frequently at one month of age (P = 0.048) (Collado et al., 2010). Similarly, in the studies by Cho et al. and Xiao et al., infants born to mothers with GDM and EGWG showed a higher relative abundance of the potentially inflammatory genera *Enterococcus* and *Prevotella* in meconium and a reduced abundance of beneficial genera like *Clostridium*, *Coriobacteriaceae*, and *Collinsella* respectively (Xiao et al., 2024; Cho et al., 2025).

### 3.4 Maternal and infant risk factors in relation to EGWG

#### 3.4.1 EGWG and GDM

The combined effect of EGWG with GDM was shown to significantly worsen dysbiosis. Cho et al. reported that GDM combined with EGWG showed a higher relative abundance of the genera with inflammatory potential (*Enterococcus* and *Prevotella*) when compared to infants of metabolically healthy mothers (Cho et al., 2025). Similarly, Xiao et al. specifically found that this concurrence of EGWG in the context of GDM resulted in the depletion of beneficial bacteria (*Clostridium, Coriobacteriaceae*, and *Collinsella*) (Xiao et al., 2024). Lastly, according to Liu et al., appropriate and excessive GWG status was one of the perinatal factors that caused significant differences in the neonatal gut microbiota, and this influence was stronger in the Control group than in the GDM group (Liu et al., 2025).

#### 3.4.2 Interaction with pre-pregnancy BMI

Collado et al. found that both maternal p-BMI and GWG were independently related to the composition of the infant gut microbiota (Collado et al., 2010). Cho et al. found taxonomic abundance displayed similar trends of variation associated with both maternal BMI and GWG groups (Cho et al., 2025). In terms of diversity, Caprara et al. found that newborns of mothers classified as obese (high p-BMI) exhibited lower alpha diversity, as also seen in EGWG women included in the study (Caprara et al., 2024).

#### 3.4.3 Other confounding factors

The observed association between maternal EGWG and infant gut dysbiosis is highly affected by several confounding factors. Delivery Mode and Perinatal Antibiotic Use constitute two of the commonest observed factors. As reported by Baumann-Dudenhoeffer et al., any microbial differences in the EGWG are often nullified or weakened by postnatal factors like delivery mode and feeding (Baumann-Dudenhoeffer et al., 2018). Kennedy et al. revealed that EGWG showed a significant effect on infant beta diversity (overall community structure), only in infants born to multiparous participants (R 2 = 0.378, p=0.0009) (Kennedy et al., 2023). Additionally, p-BMI, which as previously mentioned, is independently related to infant microbial composition and GDM, acts synergistically with EGWG to severely exacerbate dysbiosis (Collado et al., 2010; Caprara et al., 2024; Xiao et al., 2024; Cho et al., 2025). Parity was also noted as a confounding factor. According to Kennedy et al. the overall impact of EGWG on taxonomic composition is crucially modulated by maternal parity. The *Bifidobacterium* increase paired with a decrease in *Bacteroides* was only observed in infants of multiparous mothers, a shift not present in infants of primiparous mothers (Kennedy et al., 2023). Finally, the Infant Diet and Postnatal Environment play a significant role too. While EGWG establishes an adverse microbiome at birth, the sustained influence of human milk exposure and weaning eventually becomes the dominant force structuring the microbial community by 12 months (Raspini et al., 2021; Song and Liu, 2023).

### 3.5 Functional, metabolic, and later-life implications

#### 3.5.1 Functional and metabolic capacity

In the study by Chu et al. the researchers found that GWG had little impact on the metagenome within the infant stool, suggesting that-despite variations in taxa- the overall metabolic activity may be preserved in infancy (Chu et al., 2017). In contrast with the finding by Chu et al., Singh et al. found that maternal metabolic status, which was highly correlated to GWG status was associated with a reduced abundance of SCFA-producing bacteria and lower fecal butyric acid in infants (Singh et al., 2020). Additionally, Collado et al. revealed that infants of mothers with EGWG exhibited a tendency toward a higher abundance of *Akkermansia muciniphila* at one month of age (36.4% vs. 15%, P = 0.095), a potential marker of altered gut barrier function (Collado et al., 2010). Lastly, one study reported that GWG overall, independently predicted a persistent, eight-month shift in the infant gut microbiome toward enriched pathways for carbohydrate degradation and the synthesis of critical vitamins. This effect was independent of major confounders, including pre-pregnancy BMI and postnatal feeding (Baumann-Dudenhoeffer et al., 2018).

#### 3.5.2 EGWG and infant growth outcomes

Collado et al. showed associations between specific microbial groups and infant weight at 6 months of age: a higher ratio of *Bifidobacterium* to the *C. coccoides* group was associated with lower infant weight at six months (r=-0.272, P = 0.070), while higher numbers of *Clostridia* (the *C. coccoides* group) were associated with higher infant weight (r=0.300, P = 0.051) (Collado et al., 2010). Additionally, Gilley et al. found that EGWG-caused lower diversity was correlated with increased Early Childhood Weight Gain at 12 months (Gilley et al., 2022).

## 4 Discussion

Our review, incorporating fecal samples form 1723 participants revealed that EGWG is a determinant of early infant gut characteristics, primarily manifesting as a reduction in alpha diversity that appears to be persistent over the first year of life. Taxonomically, this is linked to a notable shift away from beneficial genera, such as *Bacteroides* and toward the colonization of opportunistic and potentially harmful taxa.

*In utero* exposure to metabolic and nutritional disturbances such as those often accompanied or exacerbated by EGWG, is detrimental to the fetus’s well-being. This exposure is thought to reduce the child’s cardiometabolic health and cause long-term neurological defects, including motor development disorders, as well as increased risk for childhood overweight and obesity (Lackovic et al., 2024). Additionally, early pregnancy BMI and GWG independently influence offspring growth patterns, even from birth, were these factors were associated with birth weight. From birth to 18 months, GWG affected infant growth adversely, with excessive GWG leading to diminished growth trajectories (Österroos et al., 2024).

It is important to make the distinction between the effect of maternal p-BMI on the infant gut microbiome from the dynamic GWG, which constitutes a separate metabolic variable along with other related maternal factors, such as maternal lifestyle and diet (Liu et al., 2025). In regards to perinatal gut microbiota composition, GWG seems to affect the metabolic environment during pregnancy, leading to different microbial composition irrelevant to p-BMI, which also affects the earliest colonization patterns in meconium (Cho et al., 2025). This perinatally defined factor has outcomes that extend into later childhood, e.g. maternal overweight p-BMI correlates with both offspring gut microbiota composition and diminished cognitive development at 36 months of age (Guzzardi et al., 2022).

In the study by Cho et al., two distinct pathways through which maternal weight status influences the neonatal gut are suggested. In regard to p-BMI, it appears to set a basal metabolic environment, affecting early colonization through sustained maternal energy balance, adipokine signaling, and inflammatory regulation. On the other hand, GWG acts as a dynamic variable, altering the intrauterine metabolic status, possibly through changes in nutrient delivery, short-chain fatty acid transfer, or altered gut permeability (Cho et al., 2025). On the contrary, Stanislawski et al. reported that, while excessive GWG was associated with differences in the composition of the maternal gut microbiota at delivery, these changes resulted in only limited differences in the infants’ gut microbiota over the first two years, suggesting maternal weight is not a major determinant compared to factors like delivery mode, or breastfeeding. Nevertheless, the presence of specific maternal taxa (e.g., those vertically transmitted) translated to increased presence in the infant, suggests that the relationship of EGWG and infant gut microbiota is at least partially mediated by maternal gut microbiota composition during pregnancy, influencing early colonization and microbiome maturation. EGWG was linked to an increase in potentially harmful groups like *C. difficile* and the *Staphylococcus aureus* group and a higher proportion of *Akkermansia muciniphila* at one month of life, which all correlated with future growth parameters (Stanislawski et al., 2017).

As previously mentioned, the influence of these maternal characteristics, including EGWG on the infant microbiome appears to diminish over time, as the infant and its gut ecosystem become exposed to other, more potent factors. Primarily nutrition, including feeding type (breast milk or formula) and the introduction of solid foods, appears to be the key shaping factor later in life, followed by environmental factors like contact with pets and relatives (Laursen et al., 2016). It is worth mentioning that most studies failed to account for the quality of the maternal diet when determining this effect, despite the dominant role that nutrition plays in shaping the microbiome. Nevertheless, some studies suggest that once delivery mode and infant feeding status (e.g., exclusive breastfeeding), are accounted for, many dietary associations lose statistical significance. Despite the overall uncertainty of evidence, some data suggest that specific macronutrients have an independent impact. For example, diets rich in beneficial factors like fermentable fiber and vegetable protein are found to be associated with more favorable microbiotal diversities, while diets high in animal protein and fats can increase risks of adverse outcomes related to the microbiomic environment they foster (Rio-Aige et al., 2025).

This study has certain limitations. Firstly, the availability of evidence was restricted, as our literature search retrieved only15 relevant studies, all of which were designed as observational cohorts (although, as per our protocol, RCTs were eligible for inclusion, none were retrieved) and are therefore susceptible to residual confounding. Secondly, significant heterogeneity among the included studies, in terms of design, data collection methods, and analysis, prevented the performance of a meta-analysis and made direct comparisons challenging. Lastly, the variability of GWG categories definition may affect the robustness of our conclusions and decrease the strength and generalizability of our findings.

## 5 Conclusion

In conclusion, EGWG is an independent defining factor of infant gut dysbiosis. It affects microbial alpha diversity for up to 12 months, changing the taxonomic composition, from *Bacteroides* and other beneficial colonies toward opportunistic/pathogenic genera. Although EGWG is as a key modifiable maternal factor linking gestational health to long-term offspring health, the literature remains scarce. More randomized studies and pathway research are needed to determine the exact effect of EGWG on infant gut microbiota and the mechanisms orchestrating this interaction.

## Data Availability

The original contributions presented in the study are included in the article/supplementary material. Further inquiries can be directed to the corresponding author/s.
